# Thermodilatometric Study of the Decay of Zeolite-Bearing Building Materials

**DOI:** 10.3390/ma14133551

**Published:** 2021-06-25

**Authors:** Michele Pansini, Angelo Cappi, Vincenzo Monetti, Enrico Di Clemente, Maurizio de Gennaro, Marco D’Amore, Rosa Buccino, Pierpaolo Santimone Nuzzi, Bruno de Gennaro

**Affiliations:** 1 INSTM Research Unit, Department of Civil and Mechanical Engineering, Università degli Studi di Cassino e del Lazio Meridionale, Via G. Di Biasio 43, 03043 Cassino, Italy; 2 CBC Group SpA, Via Casellina 269, 41058 Vignola, Italy; a.cappi@cbcgroup.it (A.C.); mauriziodegennaro1940@gmail.com (M.d.G.); marcodamore2013@libero.it (M.D.); r.buccino@cbcgroup.it (R.B.); p.santimone@cbcgroup.it (P.S.N.); 3 Dipartimento di Scienze della Terra dell’Ambiente e delle Risorse, Università di Napoli Federico II, Via Cinthia 26, 80126 Napoli, Italy; monetti@unina.it (V.M.); enrico.diclemente@unina.it (E.D.C.); 4 ACSLabs-Applied Chemistry Labs, Department of Chemical, Materials Engineering and Industrial Production, University of Naples Federico II, P.le V. Tecchio 80, 80125 Naples, Italy; bruno.degennaro@unina.it

**Keywords:** zeolite-bearing rock, plaster, thermodilatometry, thermal expansion, dehydration, decay

## Abstract

Six zeolite-bearing rocks, often used as building materials, were analyzed by thermodilatometry, together with a rock not bearing zeolites and a plaster covering a containing wall made of zeolite-bearing dimension stones, up to 250 °C. The main results obtained were the following: (i) the zeolite-bearing rocks exhibited very small, if any, positive variation of ΔL/L_o_ (%) up to about 100 °C, whereas they more or less shrank in the temperature range 100–250 °C (final values ranging from −0.21 to −0.92%); (ii) the rock not bearing zeolites regularly expanded through the whole temperature range, attaining a final value of 0.19%; (iii) the plaster showed a thermodilatometric behavior strongly affected by its water content. Obtained results were interpreted based on plain thermal expansion, shrinkage by dehydration, cation migration and thermal collapse of the zeolitic structure. The decay of the zeolite-bearing building materials was essentially related to: (i) the large differences recorded in the thermodilatometric behavior of the various rocks and the plaster; (ii) the different minerogenetic processes that resulted in the deposition of the various zeolite-bearing rocks.

## 1. Introduction

Natural zeolite-bearing rocks are widespread in many countries of the world in deposits of large dimensions that exhibit potential for practical applications [1]. Consequently, a number of valuable studies concerning the possible applications of natural zeolite-bearing rocks have appeared in recent decades. In particular, natural zeolite-bearing rocks have already been tested in environmental protection studies [2,3,4,5,6] for ceramization purposes aiming at tiles production [7,8,9], for producing lightweight aggregates to be used for concrete manufacture [10,11,12], in oenological refining processes [13], in the immobilization of radionuclides [14], as an additive in animal diets [15], as drug carriers [16], and in solar energy storage applications [17]. However, the use of natural zeolite-bearing rocks as building stones or building materials became a practice decidedly earlier than all the previous interesting applications, as the large number of ancient artistic and monumental building, located in many countries of the world, testify [18]. Such a widespread use is partly related to a group of technological peculiarities of natural zeolite-bearing rocks, which make them very valid building materials [19], and partly to their undoubtable beauty. Natural zeolite-bearing rocks, as with every building material, are prone to weathering. This issue, together with their frequent use in artistic and monumental constructions, has triggered many studies concerning their decay and restoration [20,21,22].

In the past years, the thermodilatometric behavior of zeolites and clays has been studied [23,24,25,26,27,28,29,30,31,32,33,34,35]. In particular, these studies have been performed on zeolite A [23,24,25,26,27,28,29], synthetic zeolite X and Y [24,28], natural natrolite, thomsonite, scolecite, heulandite, clinoptilolite, stilbite [30,31], natural chabazite [32], and various phillipsite and chabazite bearing tuffs [33]. In these works, the thermodilatometric behavior of the various zeolites or zeolite-bearing materials have been found to be related to: (1) shrinkage by dehydration; (2) thermal expansion upon heating; (3) thermal collapse of the zeolitic structure; and (4) possible cation migration from one site to another. These works have allowed the conclusion to be drawn that the thermodilatometric behavior of zeolites is strongly affected by their framework type and by their chemical composition (both the Si/Al ratio and extra framework cations).

Physical weathering of zeolite-bearing building materials appears strongly related to their thermodilatometric behavior. Although this fact is quite evident, the previously cited studies, both those concerning the weathering of zeolite-bearing building materials, as well as those concerning their thermodilametric behavior, have not seemed to take into account the interconnected relationships occurring between these two phenomena. Thus, this last one is the main goal of this work. In the present study, some zeolite-bearing rocks, which are often used as building materials, were subjected to thermodilametric investigation. Moreover, such investigation was also performed on a building material obtained from a rock, which, although it exhibited a chemical composition similar to the zeolite-bearing rocks, did not contain any natural zeolites, for the sake of comparison. Then, thermodilatometric studies were performed both on cylindrical specimens directly cut from the original rocks and on cylindrical compacts obtained by pressing powders derived from grinding these same rocks, in order to ascertain the extents to which the different natures of the samples subjected to this kind of investigation could affect their final results. Finally, the results of the thermodilatometric runs performed on the zeolite-bearing rock specimens were compared with the results of the thermodilatometric test performed, under the same experimental conditions, on a sample of a plaster covering the zeolite-bearing building stones of a retaining wall located in Parco Grifeo (in the center of Naples), built in the early 1950s.

As already established, the zeolite-bearing rocks subjected to thermodilatometric investigation in this work are used as building materials and come from huge outcrops, which makes their cost very low. The features of such zeolite-bearing rocks, as well as those of the outcrops from which they originate, are reported in the literature [36,37,38,39,40,41,42,43].

## 2. Materials and Methods

Seven different rocks that are commonly used as building materials were tested. These rocks are identified by their names followed by their site of origin and assigned a number as follows:

1. Campanian Ignimbrite from Dugenta (outskirts of Caserta, Campania region, Southern Italy);
2. Ignimbrite from Sorano (outskirts of Grosseto, Toscana region, Central Italy);
3. Campanian Ignimbrite from Comiziano (outskirts of Naples, Campania region, Southern Italy);
4. Neapolitan Yellow Tuff from Chiaiano (outskirts of Naples, Campania region, Southern Italy);
5. Neapolitan Yellow Tuff from Toledo subway station (center of Naples, Campania region, Southern Italy);
6. Sardinian Epiclastite from Anela (outskirts of Sassari, Sardinia region, Insular Italy);
7. Gray Campanian Ignimbrite from Faicchio (outskirts of Benevento, Campania region, Southern Italy).

These volcanoclastic rocks, whose mineralogical composition is reported in Table 1, are related to volcanic activities that resulted in large deposits with high amorphous components (volcanic glass). Such volcaniclastic rocks underwent a secondary minerogenetic process that led to the crystallization of zeolite phases from the original glass fraction. 

Such secondary processes of mineral transformation led to the final formation of various amounts of different products, in particular:

1. In the cases of rocks 1, 2 and 3 (the various ignimbrites), these processes led to the crystallization of a higher amount of chabazite, together with a lower amount of phillipsite and a small amount of analcime.
2. In the cases of rocks 3 and 4 (the Neapolitan Yellow Tuff), the phillipsite and chabazite contents are usually similar, although, in some cases, the former largely prevailed on the latter (see rock 4, TG Chiaiano); moreover, in the Neapolitan Yellow Tuff, analcime is present only to very low extent, if any.
3. In the case of rock 6 (the Sardinian epiclastic rock), the circulation of hydrothermal fluids through the riodacitic and ryolithic volcanic glass precursor resulted in the crystallization of clinoptilolite and smectite, together with a small amount of crystobalite and, sometimes, opal CT [39].
4. In the case of rock 7 (the Gray Campanian Ignimbrite), the high emplacement temperatures determined the welding of the glass shards and transformation of the original alcaly trachytic glass into the feldspars that represent its main constituent (more than 90%) [41].

Table 2 reports the chemical compositions (in the form of oxides) of the various rocks. Some particular analyses were performed by ICP-OES, using both a Perkin-Elmer Optima 2100 DV ICP-OES and Perkin-Elmer AVIO 200 apparatus (Waltham, MA, USA), after digestion, under microwave-induced heating (Perkin-Elmer Multiwave 3000 oven) of a weighted amount of sample in a mixed HCl, HNO_3_ and HF solution, followed by the addition of H_3_BO_3_ to attain fluoride complexation [49,50].

The typical trachytic composition of rocks coming from Phlegraean Fields (the various Campanian Ignimbrites and Neapolitan Yellow Tuff), ranging from latite to trachyte [40,41,43], is evident. Such composition appears similar to the one of the Ignimbrite from Sorano [45]. Unlike rocks 1–5, rock 6 (the Sardinian Epiclastite from Anela) exhibits a different chemical composition, resulting in a substantially different volcanic precursor with a composition varying from dacitic to rhyolitic. On the whole, the different chemical compositions of the various volcanic precursors favor the crystallization of zeolites of intermediate acidity, such as phillipsite and chabazite, in the volcanic emissions of Campania and Tuscany, whereas the formation of decidedly more acidic zeolites, such as clinoptilolite, may be considered in the volcanic emissions of Sardinia.

Table 3 reports the open porosity values of the rocks. These values were measured using a helium Multi Volume Pycnometer 1305 Micromeritics (±2%) [54] on cylindrical specimens of rocks, prepared using a water lubricated columnar drill (d = 26 mm). The surfaces of these cylindrical specimens were smoothed using a circular saw comprising a disc coated with diamond powder. The dimensions of the cylindrical specimens were as follows: h (height) ranging from 20 to 27 mm, and d (diameter) ranging from 25.6 and 25.9 mm. The cylindrical specimens were washed with distilled water and dried at about 70 (±5) °C until their recorded masses operations did not vary by less than 0.1% across two successive specimen weight.

Together with the cylindrical specimen of the seven previously described rocks, a thermodilatometric test was also performed on a specimen of plaster covering a containing wall located in Parco Grifeo (in the center of Naples), made of zeolite-bearing dimension stones. As far as the plaster is concerned, a prismatic sample with dimensions 11.1, 13.1 and 1.65 mm was obtained. The total volume of the specimens was calculated after determining dimensions using a precision gauge. Their apparent specific weight (γ, g/cm^3^) was calculated as the ratio of the mass of the dry specimen to its total volume, previously defined; their real specific weight (γ_r_, g/cm^3^) was calculated as the ratio of the mass of the dry specimen to the volume of its lone solid part. Their open porosity percentage (P) was calculated using the following formula:

P = (γ_r_ − γ) · 100/γ_r_ (1)

The values of open porosity reported in Table 3 appear related to the type of rock considered. Actually, the two Neapolitan Yellow Tuff rocks exhibit the highest porosity values (about 50–52%), the various Ignimbrites exhibit intermediate porosity (about 46–48%) and the Epiclastite exhibits the lowest porosity (about 36%). The porosity of the plaster, determined as the average of two determinations, only slightly different in the computed values, is higher than the Sardinian Epiclastite (rock 6), slightly lower than the various Campanian Ignimbrites (rocks 1–3), and substantially lower than the Neapolitan Yellow Tuff (rocks 4 and 5). 

Thermodilatometric analyses were performed for all the materials studied using cylindrical specimens obtained through the same methodology already described, utilizing in this case a columnar drill (d = 10 mm). The dimensions of the cylindrical specimens were as follows: h (height) ranging from 15 to 26 mm, and d (diameter) ranging from 9 and 9.5 mm. The cylindrical specimens were washed with distilled water and dried at about 70 (±5) °C until their recorded masses did not vary by less than 0.1% across two successive specimen weight operations.

The original rocks were ground so as obtain a sand. Such sands were put in cylindrical molds and pressed at 120 bar using a Mignon SSN/EA press, so as to obtain cylindrical compacts (h = 8 ÷ 9.4 mm, d = 10 ÷ 20 mm).

Both the cylindrical specimens directly cut from the original rocks or plaster and the cylindrical compacts obtained by pressing the ground original rocks were tested via thermodilatometry at ≤250 °C, according to the following procedure. The cylindrical specimens or compacts were put into the measuring chamber of a Thermomechanical Analyzer NETZSCH TMA 402F3 TMA402F3A-0178-M (Selb, Deutschland), whose initial temperature was set at 30 °C, and left therein for about 30 min to ensure a uniform temperature throughout the whole volume of the specimens or compacts. The temperature was then raised at a 10 °C/min heating rate in air, and the percentage variation of length of the cylinders (ΔL/L_o_ (%)(±0.01%)) was recorded as a function of the temperature. The same cylindrical specimen directly cut from a plaster used in the described thermodilatometric analysis was also subjected to a thermodilatometric test at a 5 °C/min heating rate and temperature ≤ 100 °C. The thermodilatometric curves were drawn by connecting the experimental points that appear on the diagrams by linear segments. Each experimental point was performed just once.

The thermogravimetric analysis (TG and DTG) of various rocks and plaster was performed up to 1200 °C using a Netzsch STA 449 F3 Jupiter Simultaneous Thermal Analyzer (Selb, Deutschland), equipped with Netzsch Proteus 6.1.0 software (V. 6.1.0, Selb, Deutschland).

## 3. Results

**Figure 1:**
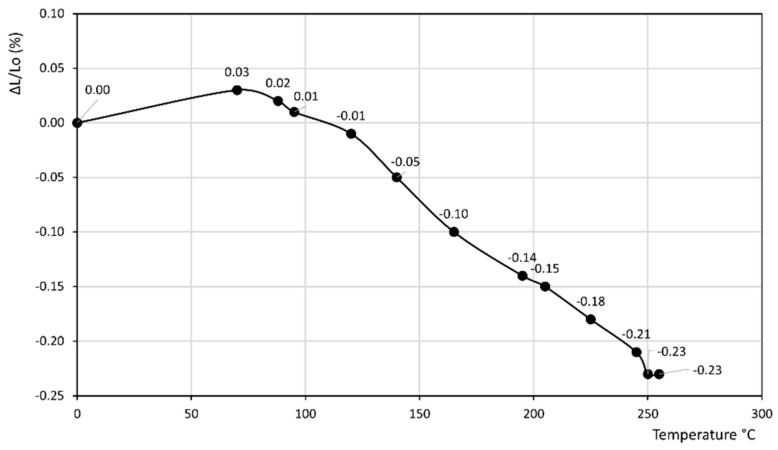
Thermodilatometric curve of rock 1, Campanian Ignimbrite from Dugenta.

**Figure 2:**
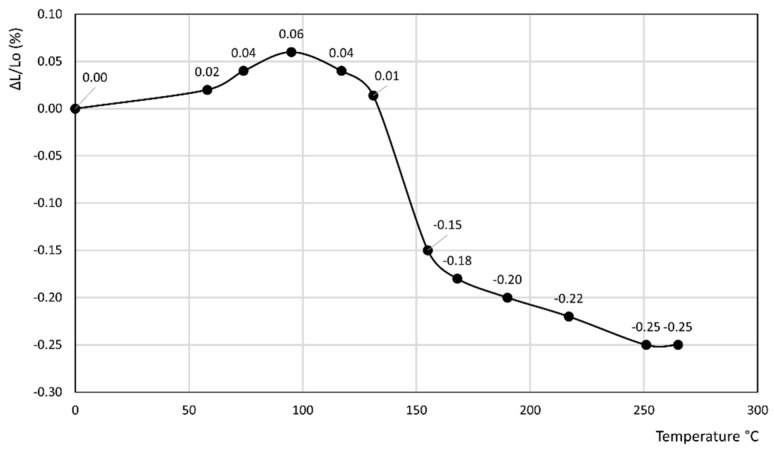
Thermodilatometric curve of rock 2, Ignimbrite from Sorano.

**Figure 3:**
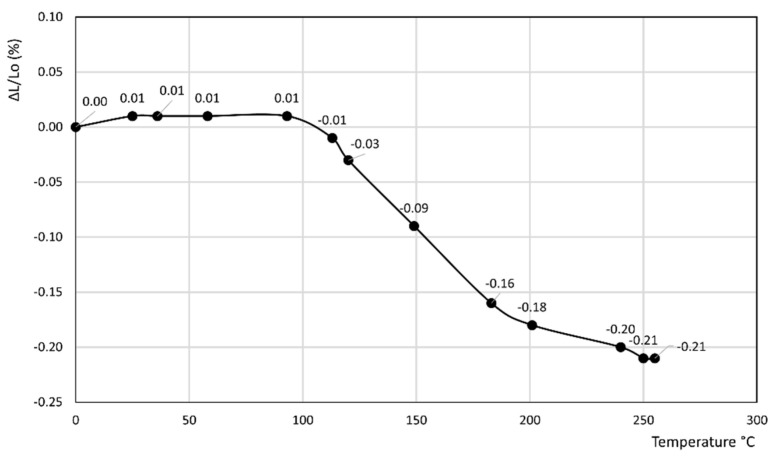
Thermodilatometric curve of rock 3, Campanian Ignimbrite from Comiziano.

**Figure 4:**
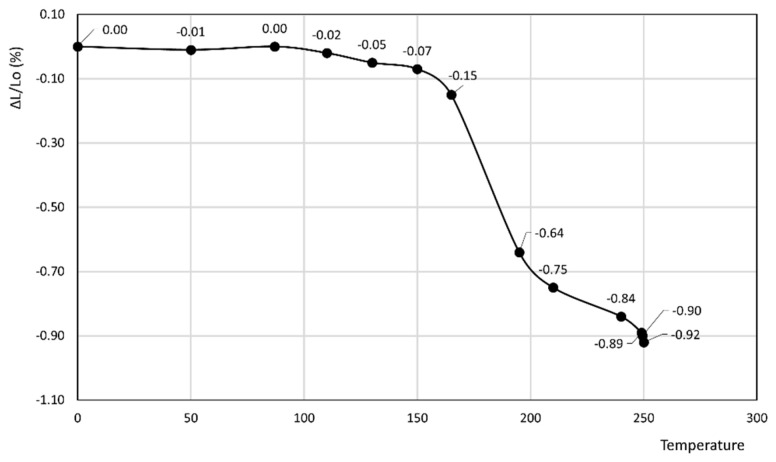
Thermodilatometric curve of rock 4, Neapolitan Yellow Tuff from Chiaiano.

**Figure 5:**
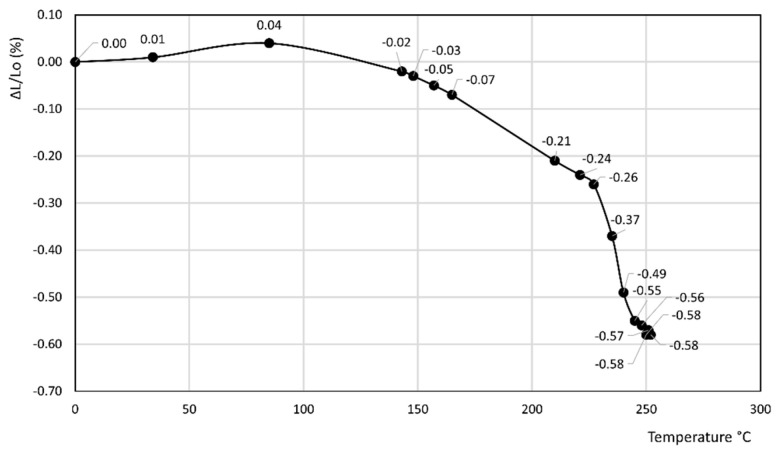
Thermodilatometric curve of rock 5, Neapolitan Yellow Tuff from Toledo subway station.

**Figure 6:**
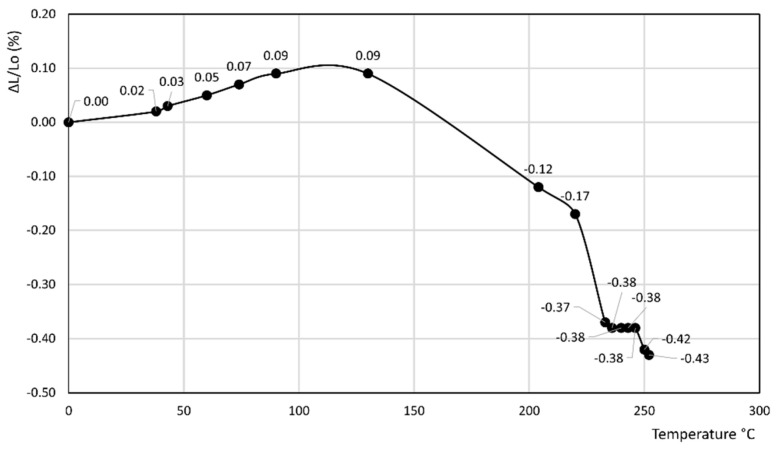
Thermodilatometric curve of rock 6, Sardinian Epiclastite from Anela.

**Figure 7:**
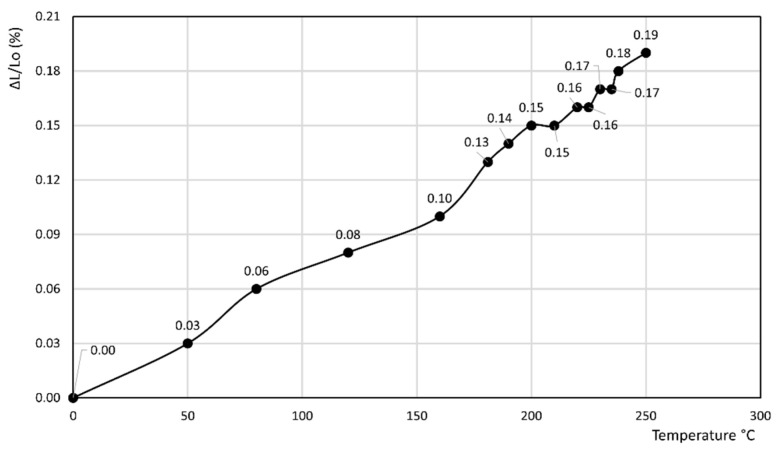
Thermodilatometric curve of rock 7, Gray Campanian Ignimbrite from Faicchio.

Figure 1, Figure 2, Figure 3, Figure 4, Figure 5, Figure 6 and Figure 7 report the thermodilatometric curve of the cylindrical specimens directly cut from the various rocks. To ensure easy comprehension, the various figures are labeled with the same number used to label the various rocks (namely, Figure 1 refers to rock 1, Figure 2 refers to rock 2 and so on). A careful comparison of the thermodilatometric curves performed on the cylindrical specimens directly cut from the various rocks with the thermodilatometric curve of the cylindrical compacts obtained by pressing the sands that resulted from grinding the original rocks (not reported) shows that both curves exhibit the same shape. In some cases (rocks 4 and 6), the numerical values of the cylinder length variation percentage (ΔL/L_o_ (%)) recorded at the various temperatures is very similar in both curves. In some other cases (rocks 1, 2, 5 and 7), a small discrepancy can be seen in the two different curves between the values recorded at homologous temperatures. Only in the case of rock 3 is this discrepancy decidedly larger than in the case of the other rocks, although it is noteworthy that the shape of the two curves remains similar. 

Figure 1, Figure 2 and Figure 3 refer to the various Ignimbrites. In the first part of these curves, the variation percentage of the cylindrical specimen’s length (ΔL/L_o_ (%)) slightly increases (from 0.01 to 0.06%) with the temperature up to about 70–90 °C. Temperatures higher than this value give rise to an evident shrinkage of the length of the specimens. In particular, it is found that: (i) the values of ΔL/L_o_ (%) decrease almost linearly with the temperature for rock 1; and (ii) the negative slope of the thermodilametric curve is more pronounced in the 90–170 °C temperature range than in the 170–250 °C range for rocks 2 and 3. Despite the slightly different trend of the various curves, the final values of ΔL/L_o_ (%) attained at 250 °C in the thermodilatometric curves of the three different Ignimbrites are similar (ranging from −0.21 to −0.25%). 

Figure 4 and Figure 5 report the thermodilatometric curves of two different specimens of Neapolitan Yellow Tuff, which, up to about 100 °C, exhibit a slightly different trend. Actually, the length of the cylindrical specimen of rock 4 remains about constant, whereas ΔL/L_o_ (%) of rock 5 attains a small maximum of about 0.04%. The cylindrical specimens of rocks 4 and 5 shrink at temperatures higher than 100 °C. In particular, as far as rock 4 is concerned, the reduction of the length of its specimen is slight in the 100–160 °C temperature range, more evident in the 160–200 °C temperature range, and finally again attenuates in the 200–250 °C temperature range, thus attaining a final value of −0.92%. As far as rock 5 is concerned, the reduction of the length of its specimen is slight in the 100–220 °C temperature range and more evident in the 220–250 °C temperature range, thus attaining a final value (−0.58%) lower than rock 4.

Figure 6 reports the thermodilatometric curve of an Epiclastite from Sardinia (rock 6). The length of the cylindrical specimen of this rock slightly increases with temperature up to about 100 °C, attaining a maximum of about 0.10%. The cylindrical specimen shrinks slightly in the 100–230 °C temperature range and evidently in the 230–250 °C temperature range, thus attaining a final shrinkage of about −0.43% at 250 °C.

The thermodilatometric curve of the Campanian Ignimbrite from Faicchio (rock 7, not bearing zeolites), reported in Figure 7, is completely different from the thermodilatometric curves previously presented. Actually, the length of the cylindrical specimen of this rock almost linearly increases at temperature up to about 250 °C, attaining a final value of ΔL/L_o_ (%) of about 0.19%.

The thermogravimetric and differential thermal analysis curves of the powder-like sample of the original zeolite-bearing rocks 1–6 (not reported) exhibit similar features. Total water loss ranges between about 11 and 14%, which is in substantial agreement with the literature data reported in Table 2. Most of this water is lost in the 20–350 °C temperature range, which is confirmed by the large spread of the endothermic peak recorded in this temperature range by the DTA curve. In particular, the dehydration process seems to start more slowly up to 70–80 °C, then seems to accelerate at higher temperatures up to 350 °C and, finally, largely slows down and attenuates at temperatures higher than this value. It must be said that in the thermogravimetric curve of the powder-like sample of the original rocks 3 and 5, a loss of water occurring at about 700 °C is evident. This loss of water is confirmed by the endothermic peak recorded in the differential thermal analysis curve at this same temperature. The thermogravimetric and differential thermal analysis curve of the powder-like sample of the lone building material not bearing zeolites (rock 7, not reported) strongly differs from those of rocks 1–6. Actually, the water loss is far smaller (2.2%, in good agreement with the literature data of Table 2), and the endothermic peak denoting dehydration is decidedly sharper in rock 7. Despite this, dehydration seems to continue even at temperatures higher than 800 °C.

**Figure 8:**
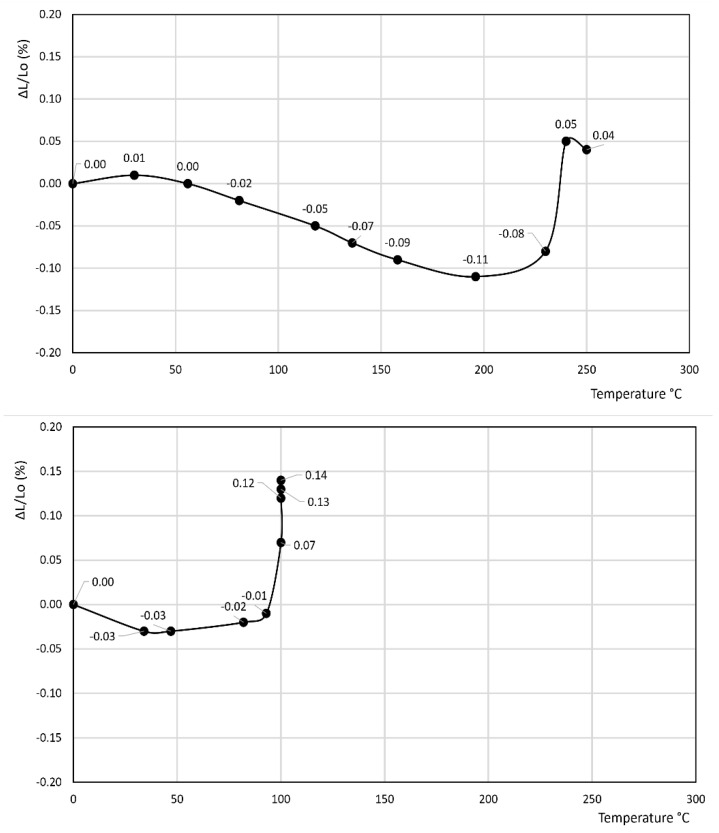
Thermodilatometric curve of the cylindrical specimen directly cut from the plaster recorded at 10 °C/min (**top** panel) and 5 °C/min (**bottom** panel).

Figure 8 reports the thermodilatometric curve of the cylindrical specimen directly cut from the plaster recorded at 10 °C/min (top panel) and 5 °C/min (bottom panel). The two curves do appear rather different:

1. In the former curve, ΔL/L_o_ (%) decreases up to 200 °C, thus attaining a minimum of about −0.11%, then rapidly increases up to about 240 °C, thus attaining a maximum of about 0.05%, and finally decreases again to a final value of 0.04% at 250 °C.
2. In the latter curve, ΔL/L_o_ (%) slightly decreases up to 40 °C, attaining a value of −0.03%, thus attaining a plateau at this value in the 40–80 °C temperature range before finally increasing to a final value of 0.13% at 100 °C.

**Figure 9:**
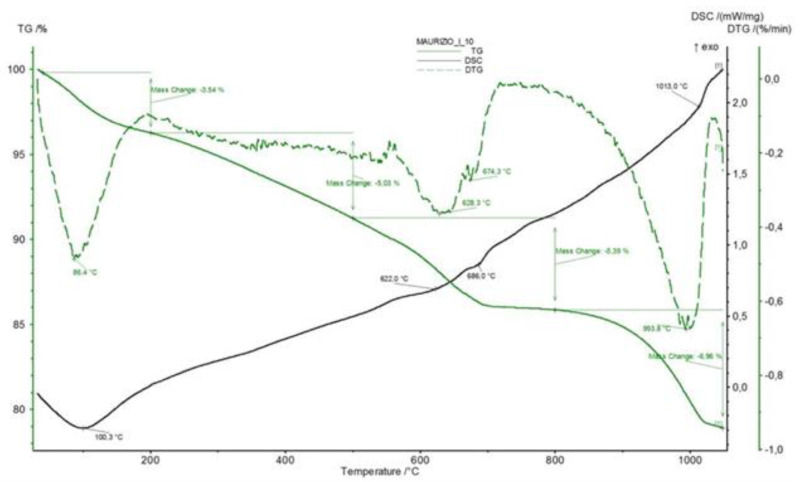
DTA and TG analysis of the plaster.

Figure 9 reports the DTA and TG analyses of the plaster. Three endothermic effects at about 86, 820 and 993 °C are recorded in the former curve. The latter curve denotes a progressive dehydration, characterized by a fundamentally linear trend, over the whole temperature range, attaining a final water loss of about 20.6%. 

## 4. Discussion

In this work, the thermodilatometric curves of cylindrical specimens cut directly from various rocks were compared with the thermodilatometric curves of cylindrical compacts obtained by pressing the sands obtained from grinding the original rocks. We ascertained the extent to which the different modalities of preparing the sample to be subjected to the thermodilatometric analysis affected the final results. It was shown that the results obtained by following the two different modalities were, in some cases (rocks 4 and 6), almost equal, while some others were similar in the nature with slightly different numerical values (rocks 1, 2, 5 and 7), and, in one case (rock 3), similar in the nature but characterized by more evident differences in the numerical values. Thus, it can be concluded that it is better to perform thermodilatometric analysis on the cylindrical specimens directly cut from rocks, as this modality warrants the most homogeneous results between the tested samples and the actual building materials. However, the results obtained via thermodilatometric analysis for the compacts obtained from pressing the sands obtained after grinding the original rocks supplied reliable results, demonstrating that the preparation of the compact was perfectly performed. Actually, the larger discrepancies recorded between the thermodilatometric analysis of the cylindrical specimen directly cut from rock 3 and the thermodilatometric analysis of the compact obtained by pressing the sand obtained by grinding rock 3 can be reasonably assumed to be an imperfect preparation of the pressed sample.

Rocks 1–3 contained chabazite as the predominant phase together with a smaller amount of other zeolites such as phillipsite and analcime, although this predominance occurred to a different extent (see Table 1). In the thermodilatometric curves of these rocks, plain thermal expansion prevailed during shrinkage resulting from dehydration in the 30–90 °C temperature range. At higher temperatures, the amount of water leaving the zeolite framework increased to such an extent that the shrinkage resulting from dehydration prevailed on the plain thermal expansion, thus giving rise to a negative slope on the curve. On the whole, the thermodilatometric curves of rocks 1–3 seem consistent with the thermodilatometric behavior of the Na-, K-, Cs-, Mg-, Ca- and Sr-chabazite [29]. This issue appears perfectly logical, as most of the cations contained in natural zeolites were those present in the various waters that contacted the parent volcanic glass or the zeolitized rock itself (Na^+^, K^+^, Ca^2+^, and Mg^2+^ are the cations usually present in hydrothermal, meteoric or hydrothermal waters) [34,35,36,37,38,39]. The limited shrinkage attained at 250 °C (ranging from −0.21 to −0.25%) may have been related to the fact that the total content of zeolites of such rocks ranged between 49 and 59% of their total mass, while the remaining components of rocks 1–3, other than zeolites, expanded as temperature increased.

Figure 4 and Figure 5 show the thermodilatometric curves of two different specimens of Neapolitan Yellow Tuff, which, up to about 100 °C, exhibited a slightly different trend. Actually, the length of the cylindrical specimen of rock 4 remained relatively constant, whereas the ΔL/L_o_ (%) of rock 5 attained a small maximum of about 0.04%. The cylindrical specimens of rocks 4 and 5 shrunk at temperatures higher than 100 °C. As far as rock 4 is concerned, the reduction of the length of its specimen was slight in the 100–160 °C temperature range, more evident in the 160–200 °C temperature range, and finally attenuated in the 200–250 °C temperature range, thus attaining the final value of −0.92%. As far as rock 5 is concerned, the reduction of the length of its specimen was slight in the 100–220 °C temperature range, and more evident in the 220–250 °C temperature range, thus attaining a final value (−0.58%) lower than rock 4.

The thermodilatometric curve of rocks 4 and 5, containing phillipsite as the predominant phase together with a smaller amount of other zeolites such as chabazite and analcime, cannot be compared to the dilatometric behavior of phillipsite, as an analysis was not performed. However, valid insight into the dilatometric behavior of rocks 4–5 might be achieved on the basis of the following:

1. Thermal behavior of phillipsite is described in [58]. In this work, the various cation forms of phillipsite were found to undergo thermal collapse at temperatures ranging from 200 to 600 °C.
2. Shrinkage resulting from dehydration in chabazite and phillipsite appears likely to be similar on account of their similar Si/Al ratio and cation populations (these zeolites originated from the alteration of the same volcanic glass by weathering) and on account of their similar framework densities (1.58 and 1.45 g/cm^3^ for phillipsite and chabazite, respectively [59]).
3. The amount of water released by zeolites at the various temperatures is strongly related to cation population, as demonstrated confidently in [60]. It is true that the cation populations of the various zeolitic phases present in the rock were not explicitly determined in that study, but the oxide analysis of the rocks (Table 2) gives us this datum, at least to a loose approximation.

On the basis of these considerations, the different behaviors up to 100 °C (the length of the cylindrical specimen of rock 4 remained about constant, whereas the ΔL/L_o_ (%) of rock 5 attained a small maximum of about 0.02%, see Section 3) may be explained by the fact that rock 4 exhibited a higher content of K^+^ and a lower content of Na^+^ and Ca^2+^ than rock 5. Actually, in this low temperature range, a large monovalent cation such as K^+^ holds zeolitic water less strongly than a smaller monovalent cation such as Na^+^, or a smaller divalent cation such as Ca^2+^ [60]. This allows low-temperature dehydration to occur to a larger extent with consequently larger shrinkage, which counterbalances the plain thermal expansion, thus giving rise to the absence of appreciable length variations of the cylindrical specimen.

The lower final value of shrinkage attained by rock 4 (−0.92%) versus rock 5 (−0.60%) may be justified by its higher phillipsite content (55% for rock 4 and 37% for rock 5), which partly undergoes thermal collapse in the temperature range 200–600 °C [58], thus giving rise to large shrinkages of cylindrical specimens.

The thermodilatometric curve of rock 6 appeared consistent with the thermodilatometric curve of Na- and K-clinoptilolite reported in [31]. Also noticeable in this thermodilatometric curve, a slight increase in the length of the cylindrical specimen (0.10%) recorded at about 100 °C can be ascribed to the fact that, in this temperature range, plain thermal expansion prevails on shrinkage resulting from dehydration. At temperatures higher than 100 °C, shrinkage caused by dehydration overwhelms plain thermal expansion, thus giving rise to a reduction of the length of the cylindrical specimen, which becomes very evident at temperatures higher than about 220 °C. The limited value of the shrinkage attained at the highest temperature (−0.43%) seems to exclude the irreversible structural changes observed in the 230–260 °C temperature range, as described by Gottardi and Galli [61] in the type I thermal behavior of clinoptilolite. In particular, such limited value of the shrinkage at 250 °C seems to indicate a type III thermal behavior of clinoptilolite, which Gottardi and Galli [61] describe as follows: “clinoptilolite undergoes continuous reversible dehydration with only a very small lattice contraction and the lattice is not destroyed by heating if not over 750 °C”. This consideration is further supported if the chemical composition of the clinoptilolite present in rock 6 is reported in the triangular diagram, which, according to Gottardi and Galli [61], defines the various thermal behavior of clinoptilolite. Actually, it falls in the portion of the diagram characterized by type III thermal behavior of clinoptilolite.

It must be noted that all of the zeolite-bearing rocks 1–6 slightly expanded or maintained a generally consistent length during heating in the low temperature range (up to nearly 100 °C), whereas shrinkage occurred more frequently in the 100–250 °C temperature range. A careful examination of the thermogravimetric curves (not reported) might explain this issue by considering how the small amount of water lost up to about 100 °C may give rise to

1. a small shrinkage, which almost exactly counterbalances the plain thermal expansion, thus resulting almost in the absence of appreciable length variations among specimens (rocks 4 and 5);
2. a small shrinkage of entity slightly lower than the plain thermal expansion, thus giving rise to slightly positive length variations among specimens (rocks 1, 2, 5 and 6).

Heating the various zeolite-bearing rocks in the 100–250 °C temperature range led to a more considerable water loss, resulting in a marked shrinkage of the specimens not sufficiently counterbalanced by plain thermal expansion and causing appreciable negative length variations among the specimens. However, it must be said that the thermodilatometric results were found to be slightly affected by the heating rate. In particular, [29] reports that increasing the heating rate of zeolite A compacts from 3 to 30 °C/min results in a slightly smaller shrinkage if the temperature is the same. This effect may be explained by considering how shrinkage is related to dehydration, which does not occur instantaneously. Thus, a higher heating rate results in a shorter amount of time for shrinkage to occur and, ultimately, in a smaller shrinkage (ceteris paribus). Thus, it could be said that 10 °C/min is a too quick of a heating rate to properly mimic the effects of environmental temperature variations on zeolite-bearing building materials. This objection, although valid in principle, does not seem to hold in practice on account of

1. the limited extent to which the variation of the heating rate affects the thermodilametric behavior of the zeolite-bearing materials;
2. the fact that the variation of the heating rate does not change the nature of the phenomena occurring [29];
3. the fact that the issue most largely affecting the weathering of zeolite-bearing building stones is the difference of thermodilatometric behavior between the plaster layer and the underlying building stones (vide infra).

As far as the thermodilatometric curve of the Campanian Ignimbrite from Faicchio (rock 7, not bearing zeolites) is concerned, the almost linear increase in the length of the cylindrical specimen (up to 0.18% at 250 °C) with increasing temperature can be explained by the plain thermal expansion not tackled by the dehydration of any zeolite. Actually, only the amorphous phase present in rock 7 may have undergone dehydration, but this led to a limited loss of water (2.2%) that was unable to produce appreciable negative length variations among the specimens (it should also be noted that a part of this 2.2% water was related to weakly bound water deriving from the humid environment where it was stored). Moreover, the progressive dehydration of the amorphous phase present in rock 7 reasonably explains the small fluctuations of this thermodilatometric curve.

The fact that the thermodilatometric curve of the cylindrical specimen directly cut from the plaster recorded at 10 °C/min up to 250 °C (Figure 8 top panel) and 5 °C/min up to 100 °C (Figure 8 bottom panel) are rather different is not surprising. The former curve was recorded by subjecting the cylindrical specimen of the plaster to thermodilatometric analysis after it was directly cut using a water-lubricated columnar drill and dried, as described in Section 2. It appears evident that, in spite of the drying procedure, the cylindrical specimen of the plaster maintained a relatively conspicuous amount of water inside itself that was related to the cutting operations and lost on heating, thus giving rise to a considerable shrinkage (0.11% at about 200 °C). Once most of this loosely bound water was lost, the plain thermal expansion overwhelmed the shrinkage by dehydration, thus giving rise to positive variations of length of the specimen that were characterized by a high value of the slope of the thermodilatometric curve. All these phenomena did not occur in the thermodilatometric curve, which was performed at 5 °C/min up to 100 °C on the same cylindrical specimen used during the previous thermodilatometric run. This issue perfectly explains the near-total absence of length variations up to 90 °C, as in this temperature range the loss of the very small amount of remaining water was almost perfectly counterbalanced the plain thermal expansion. At temperatures > 90 °C, the amount of water present in the cylindrical specimen of the plaster was too low to affect as a result of dehydration during the plain’s thermal expansion, thus giving rise to positive length variations of the specimen itself. This interpretation appears confirmed by the TG curve of the plaster (Figure 9), as the water loss recorded at the minimum of the thermodilatometric run (about 3.5% at 200 °C) does not appear sufficient to have justified such a large contraction of the plaster specimen’s length (TG analysis was performed on a powdered sample of plaster that did not contain the loosely bound water derived from the cutting operations of the cylindrical specimen).

**Figure 10:**
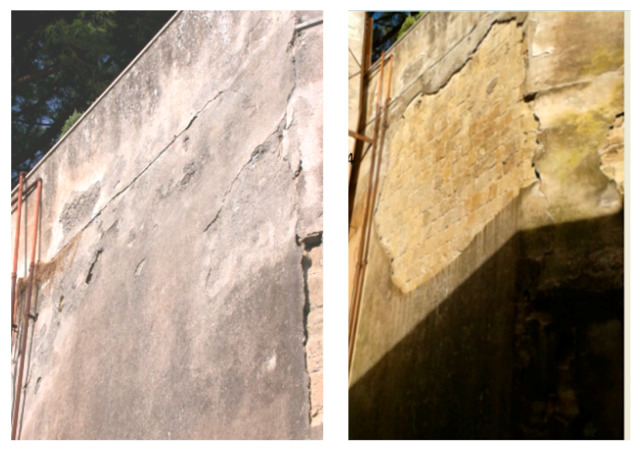
Retaining wall built in Neapolitan Yellow Tuff dimension stones in 1957, coated by a layer of plaster (Parco Grifeo, center of Naples). The picture on the left portrays the wall as it appeared in 2011, whereas the picture on the right portrays the same wall as it appeared in 2014 after the separation of the plaster (picture by M. de Gennaro).

The two thermodilatometric curves of Figure 8 appear very meaningful, as they suggest that the thermodilatometric behavior of a plaster may vary completely according to the various conditions in which it may be found. Actually, a plaster dampened by rain very likely expands more depending on the strength of the rain. This same plaster dampened by rain shrinks more as the sun becomes warmer and the wind that dries it becomes more violent. Clearly, the zeolite-bearing rocks underlying the plaster in this study were not prone to the same harsh dilatometric variations experienced by the plaster itself, as they were not exposed to weather variations. Thus, the zeolite-bearing rocks slightly expanded or shrunk, according to the thermodilatometric curves recorded. Finally, the large differences between the thermodilatometric behavior of the zeolite-bearing rocks and the plaster resulted in the occurrence of a stress state between them. Such a stress state, repeated many times as time goes by, can lead to the separation of the plaster from the underlying zeolite-bearing material, as shown in Figure 10, which concerns a retaining wall built in Neapolitan Yellow Tuff dimension stones coated by a layer of plaster (Parco Grifeo, center of Naples). Moreover, the thermodilatometric behavior of the plaster appears to be significantly different from that of rock 7 (the only one not bearing zeolites). this suggests that the separation of the plaster from the underlying unzeolitized rock will also occur, although to a different extent and with different modalities. However, a possible objection that could be raised to this interpretation is the minimal length variation of the various cylindrical specimens of zeolite-bearing rocks at temperatures < 100 °C (up to 0.09%). Despite such a small entity, it should be borne in mind that the weathering of building materials only occurs over tens of years. With such a lag of time, the small differential dimensional variations a the zeolite-bearing rocks and the overlying layer of plaster are repeated a huge number of times, thus giving rise to the decay phenomena described for the building materials.

It must be also noted that it does not appear possible to further explain the three endothermic effects recorded in the DTA curve of the plaster, as it is not known how the material was manufactured. The only consideration that can be drawn is that the effects are related to the dehydration of the material’s various constituents. Moreover, the different heating rates of the thermodilatometric runs to which the cylindrical specimen of the plaster was subjected appear to have played a secondary role in determining the features of the thermodilatometric curve itself.

**Figure 11:**
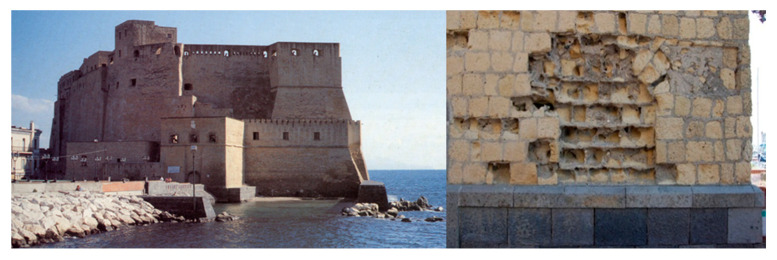
Example of the marked physical weathering of facia vista Neapolitan Yellow Tuff dimension stones, consisting of a marked consumption of their exposed surface assuming a typical concave shape (Castel dell’ Ovo, center of Naples, left: whole building, right: physical weathering; picture by A. Langella).

It must be pointed out that zeolite-bearing rocks are often used facia vista, namely, the bare prismatic dimension stones are not covered by a layer of plaster in order to expose their warm and magnificent range of colors ranging from dark yellow to ochre and reddish and light brown. This is the case for many artistic and monumental buildings (Maschio Angioino Castle, Dell’Ovo Castle and Sant’Elmo Castle, Basilica of Santa Chiara, Basilica of San Domenico Maggiore, Chapel Pappacoda, Academy Palace of Fine Arts and Grenoble Palace in the center of Naples town). As far as this use of zeolite-bearing materials is concerned, it was found that the dimension stones of Neapolitan Yellow Tuff are very prone to a particular form of physical weathering (related to temperature and, thus, dimensional variations) consisting of a marked consumption of their exposed surface assuming a typical concave shape (Figure 11). Heap et al. [60] observed this form the physical weathering in Neapolitan Yellow Tuff dimension stones in particular, whereas they found it was absent in zeolite-free tuffs. On the basis of this observation, they ascribed the phenomenon to water saturation by imbibition, resulting in the more marked expansion of zeolite and clay phases present therein with a consequent stress state that strongly affected the mechanical properties of the Neapolitan Yellow Tuff dimension stones.

**Figure 12:**
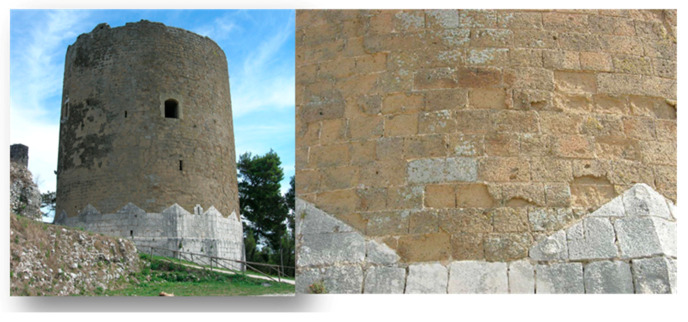
Example of the moderate physical weathering of facia vista Campanian Ignimbrite dimension stones (Mastio of the Caserta Vecchia Castle, Caserta Vecchia, **left**: whole building, **right**: physical weathering; picture by A. Langella).

This interpretation appears very likely. However, water saturation resulting from imbibition does not appear to be the main cause of the weathering of the Neapolitan Yellow Tuff, as total water saturation of dimension stones was infrequently observed. Moreover, it was found that, unlike the Neapolitan Yellow Tuff, other zeolite-bearing materials did not appear prone to this kind of physical weathering to the same extent, although in some cases, such as the Campanian Ignimbrite, the zeolites contained therein (phillipsite, chabazite and analcime) were the same (Figure 12).

It is difficult to interpret this issue, as the chemical and mineralogical nature of the two zeolite-bearing rocks are similar (see Table 1 and Table 2). Furthermore, they derive from minerogenetic processes involving volcanoclastites connected to the activity of the same volcanic complex, and the main outcrops of these two zeolite-bearing rocks are very similar to each other [39,41,43]. A possible explanation for their differing resistance to physical weathering would be the different minerogenetic processes leading to the formation of the two different rocks. Actually, it appears possible that the deposition of the Campanian Ignimbrite occurred at a temperature close to the glass transition temperature of the rock. This could be responsible for a more or less partial welding of the glass shards, resulting in good mechanical properties and enhanced resistance to weathering that originated from dimensional variations related to temperature fluctuations and, partially, water saturation, in the Campanian Ignimbrite. If the temperature of deposition of the Neapolitan Yellow Tuff were lower than that of the Campanian Ignimbrite, and thus decidedly lower than the glass transition temperature of the rock, the welding of the glass shard forming the rock itself would occur to a far lower extent. This issue would satisfactorily explain the fact that, to a large extent, the expansion of the zeolite and clay phases present in the Neapolitan Yellow Tuff affected the mechanical properties of this zeolite-bearing rock rather than the ones of the Campanian Ignimbrite, thus justifying the large difference recorded in the resistance that the two rocks exhibited to this form of physical weathering [61,62,63].

## 5. Conclusions

The results of this thermodilatometric investigation, performed on various building materials, including zeolite-bearing rocks as well as a plaster, seems to indicate that the separation of the plaster from the underlying building materials will ultimately occur both for zeolitized or unzeolitized rocks. This adverse event appears the logical consequence of the stress state created between the plaster layer and the underlying building materials, owing to their different thermodilatometric behaviors. Evidently, such separation gives rise to: (i) unpleasant aesthetic effects visually; and (ii) further decay of the underlying building materials, which have already been shown not to be especially resistant to weathering [61,62,63]. It appears to be of crucial importance to carry out further measures aimed at hindering the occurrence of such separations, in order to avoid both the unpleasant aesthetic effects and the early decay resulting from the weathering of the building materials. In the opinion of the authors of this work, one such measure would be to lay a coat of polymeric resin over the outer part of the plaster. This could be carried out before separation of plaster from the zeolite-bearing dimension stone has occurred, and after a proper preparation of the dimension stone surface has been performed. In such a case, the polymeric resin would largely reduce the wettability of the pores of the plaster, and make them essentially hydrophobic. As a consequence, the plaster, even when exposed to a very hard rain, would retain a far smaller amount of loosely bound water, which appears the main factor responsible for continuously and significantly changing the dilatometric features of the plaster itself. Creating a more stable thermodilatometric behavior of the plaster seems to be a possible means of reducing the differential variation of dimensions between the plaster itself and the underlying building materials, thus reducing the stress state between them and the consequences of their separation.

Laying a coat of a polymeric resin seems to be a valid measure by which to increase resistance to physical weathering of the zeolite-bearing dimension stones, even in cases where they were used facia vista. In such cases the reduced wettability of the pores of the zeolite-bearing rocks will also allow a smaller amount of rainwater to soak the zeolite-bearing dimension stones and consequently reduce expansion of the zeolite and clay phases present therein. Thus, it will enhance resistance to physical weathering originating as a result of water imbibition and temperature fluctuations.

## Figures and Tables

**Table 1:** Mineralogical composition of studied pyroclastic rocks.

Sample	Biotite	Feldspars	Clino-Pyroxene	Cristobalite	Clinoptilolite	Chabazite	Phillipsite	Analcime	Quarz	Amorphous and Clays	Ref.
**1 IC Dugenta**	3 (±1)	21 (±3)	4 (±1)	-	-	34 (±2)	16 (±2)	tr	-	22 (±4)	[44] ^$^
**2 TF Piandirena**	2 (±2)	16 (±3)	5 (±1)	-	-	52 (±4)	4 (±1)	3 (±1)	-	18 (±2)	[45] ^$^
**3 IC Comiziano**	1 (±1)	30 (±5)	5 (±1)	-	-	27 (±2)	21 (±1)	1 (±1)	-	15 (±2)	[45] ^$^
**4 TG Chiaiano**	0, 2	23 (±4)	-	-	-	8 (±2)	55 (±2)	3 (±1)	-	12 (±2)	[46] ^$^
**5 TG Toledo**	tr	13 (±2)	-	-	-	30 (±1)	37 (±2)	3 (±1)	-	17 (±2)	[47] ^$^
**6 CL Anela**	2 (±1)	10 (±2)		6 (±2)	58 (±2)	-	-	-	7 (±1)	17 (±4)	[48] ^$^
**7 IC Grigia Faicchio**	1 (±2)	93 (±3)	-	-	-				-	6 (±2)	[41]

^$^ These data refer to technical reports or degree/PhD theses in the possession of Prof. M. de Gennaro, who will make them available upon request.

**Table 2:** Chemical composition of studied pyroclastic rocks.

Sample	SiO_2_	TiO_2_	Al_2_O_3_	Fe_2_O_3_	MnO	MgO	CaO	Na_2_O	K_2_O	P_2_O_5_	LOI ^§^	Total	TG ^§§^	Ref. Bib.
**1 IC Dugenta**	54.80	-	15.90	3.65	0.12	1.18	4.57	1.25	5.24	0.13	12.58	99.42	14.20	[51]
**2 TF Piandirena**	50.89	0.61	17.43	5.21	-	2.29	6.39	0.96	6.04	-	10.70	100.52	11.61	[45] ^$^
**3 IC Comiziano**	54.52	0.45	15.19	4.00	0.17	0.88	4.14	1.04	6.98	0.10	12.53	99.88	10.21	[52] ^$^
**4 TG Chiaiano**	52.08	-	16.52	3.50	-	1.02	2.71	2.06	8.67	-	13.72	100.28	13.47	[53] ^$^
**5 TG Toledo**	50.70	-	16.97	3.47	0.09	1.23	3.63	2.60	7.21	0.12	14.17	100.50	14.32	[54]
**6 CL Anela**	65.62	-	12.88	0.04	-	1.08	3.22	1.32	1.94	-	13.09	100.00	12.09	[55] ^$^
**7 IC Grigia Faicchio**	60.23	0.45	18.65	4.05	0.22	0.54	1.87	4.56	7.54	0.11	1.85	100.08	2.40	[41]

^$^ These data refer to technical reports or degree/PhD theses in the possession of Prof. M. de Gennaro who will make them available upon request. ^§^ Loss on ignition. ^§§^ Weight loss percentage recorded by the thermogravimetric (TG) curve.

**Table 3:** Open porosity (P) of the various rocks and plaster.

Sample	Open Porosity (%)	Reference
**1 IC Dugenta**	48.02	[56] ^$^
**2 TF Piandirena**	48.54	[56] ^$^
**3 IC Comiziano**	45.69	[56] ^$^
**4 TG Chiaiano**	52.25	[56] ^$^
**5 TG Toledo**	50.01	[56] ^$^
**6 CL Anela**	35.87	[56] ^$^
**7 IC Grigia Faicchio**	48.82	[56] ^$^
**Plaster**	44.63	[57] ^$^

^$^ These data refer to technical reports or degree/PhD theses in the possession of Prof. M. de Gennaro who will make them available upon request.

## Data Availability

The data reported in this article concerning the experimental part containing the results of the thermodilatometric tests are original and produced at the thermodilatometry laboratory of Soc CBC Group of Vignola (MO) Italy. Chemical data have been reported from scientific publications available on international journals whose references are given below. Mineralogical: porosimetric data and chemical analysis reported as: “Italiana Zeolite (CBC Group Company) Record, Clinosa”, refer to reports of analysis made by Research Laboratories of Italian Universities are available on request from the co-author Prof. Geol. Maurizio de Gennaro responsible for research activities of raw materials of Soc. CBC Group. Data are not publicly available due to commercial confidentiality.
